# Temporal Changes in Indicators of Testicular Dysgenesis Syndrome in Labrador and Golden Retrievers

**DOI:** 10.3390/ani15142073

**Published:** 2025-07-14

**Authors:** Thomas Lewis, Rachel Moxon, Gary C. W. England

**Affiliations:** 1 Guide Dogs National Centre, Warwickshire CV33 9WF, UK; rachel.moxon@guidedogs.org.uk; 2 School of Veterinary Medicine and Science, University of Nottingham, Leicestershire LE12 5RD, UK; gary.england@nottingham.ac.uk

**Keywords:** semen, motility, cryptorchid, hybrid vigour, breed, testicular

## Abstract

Temporal changes in testicular traits have been reported in both humans and dogs. Analysis of % living sperm and motility from semen collections from 186 Labrador Retrievers and 113 Golden Retrievers between 2006 and 2023, and of incidents of cryptorchidism in over 15,000 dogs of the same breeds and crosses born between 1994 and 2023 was undertaken to determine influential factors. A general temporal increase in incidence of cryptorchidism masked significant differences in the trend between breeds, which persisted after accounting for genetic and litter effects. The incidence in the F1 cross was significantly lower than in either pure breed, implying hybrid vigour. The semen traits were both moderately repeatable within individuals, but this belied breed differences in its composition; for both traits, only the heritability was significantly greater than zero in the Golden Retriever, while only the permanent environment effect was present in Labrador Retrievers. There were significant negative temporal trends in Golden Retrievers for both semen traits, but not in Labrador Retrievers; significant negative effects of age (except on % motility in Labrador Retrievers); and significant negative effects of a diagnosis of benign prostatic hyperplasia on both traits in both breeds. These results reveal complex breed by environment interactions in traits related to testicular form and function.

## Simple Summary

Changes in testicular traits over time have been reported in both humans and dogs. Semen quality data for 186 Labrador Retrievers and 113 Golden Retrievers between 2006 and 2023, and incidents of cryptorchidism in over 15,000 dogs of the same breeds and crosses born between 1994 and 2023 were examined to determine changes over time, genetic features, and breed variations. The semen traits (fast, forward progressive motility, and percentage normal live sperm) were both moderately repeatable within individuals, but there were breed differences: For both traits, only the heritability was significantly greater than zero in Golden Retrievers, while only the permanent environment effect was present in Labrador Retrievers. There were significant negative changes over time in Golden Retrievers for both semen traits, but not in Labrador Retrievers; significant negative effects of age (except on motility in Labradors); and significant negative effects of a diagnosis of benign prostatic hyperplasia on both traits in both breeds. A general increase in incidence of cryptorchidism was present with differences between breeds, which remained after accounting for effects of genetics and litter. The incidence in the F1 cross was significantly lower than in either pure breed, suggesting improvements in crossbred offspring. These results reveal complex breed x environment interactions in traits related to testicular form and function.

## 1. Introduction

Testicular dysgenesis syndrome (TDS) has a genetic and/or environmental cause and encompasses several male reproductive disorders, including poor semen quality, testicular cancers, hypospadias, and cryptorchidism [1,2,3]. These problems are reportedly increasing globally and may be a cause of concern for future reproductive potential in some species [1,4,5,6,7]. Both declining semen quality and testicular dysgenesis syndrome have been associated with environmental chemicals, and certain chemicals have been demonstrated to be negatively associated with testicular development and function, and with semen quality [8,9,10].

A temporal decline in semen quality has been described in many species including dogs, bulls, horses, and humans [11,12,13,14]. In dogs, a decline in motility of 30% over 26 years from 1988 to 2014 has been reported. Alongside this, an increasing trend in the incidence of cryptorchidism, a marker of testicular dysgenesis, was reported over the same time period [11]. It is plausible that, should traits relating to semen quality be heritable in the dog, genetic selection may be useful in dog breeding programmes to mitigate some of the risk of the observed decline in semen quality. Previous work on semen quality heritability has shown moderate and high estimates for some traits in other species, including chickens [15,16,17], pigs [18,19], horses [20,21], goats [22], and cattle [23,24,25]. However, estimates of heritability are specific to the population in which they are calculated, and will differ according to breed, species, and model [21,23,26]. Nevertheless, there is little evidence relating to the heritability of semen quality in dogs [27].

In the same assistance dog population used by Lea et al. [11] to report temporal trends in semen quality, a simple preliminary analysis conducted for 48 dogs suggested weak correlations between parents and offspring for five measured semen parameters [28]. However, the study was limited by the small numbers of dogs and did not account for the influence of other non-heritable factors.

The aim of the present study was to utilise more recent detailed pedigree and phenotypic records pertaining to testicular dysfunction from the same managed assistance dog population to determine and characterise temporal trends, genetic features, and breed variations in semen quality and cryptorchidism.

## 2. Materials and Methods

### 2.1. Semen Quality Traits

Records of routine semen evaluations from samples collected between 1 January 2006 and 31 December 2023 from Labrador and Golden Retriever stud dogs in an assistance dog breeding programme were collated. Semen collections and evaluations were conducted as previously described [28] and carried out by four reproductive specialists who were all trained and regularly evaluated by one of the authors (G.C.W.E.). Semen evaluation records included dog identity number, breed, date of birth, date of collection/evaluation, % total normal live sperm (TNLS), and % of sperm with fast, forward, progressive motility (MOT). Motility was subjectively assessed by placing a 25 μL drop of undiluted semen onto a warmed glass microscope slide; the percentage of sperm with fast, forward, progressive motility was estimated to the nearest 5%. The numbers of live and dead spermatozoa and sperm morphology were examined by counting 100 spermatozoa on a nigrosin/eosin-stained smear using classification as previously described [28]. The variety, but low specific incidences, of morphological abnormalities led to the decision not to analyse individual morphological variations of sperm. The percentage of morphologically normal live sperm was recorded. Records were filtered to remove samples that were collected within seven days of a previous sample, were from a dog of aged 120 months or older, or were from dogs of breeds other than Labrador and Golden Retrievers.

Further information on dogs with semen evaluation records was extracted from internal databases. For each dog, the inbreeding coefficient was calculated (from entire pedigree data according to Meuwissen and Luo [29]), and for each semen sample, the presence/absence of a diagnosis of benign prostatic hyperplasia (BPH) was retrieved. BPH was diagnosed upon clinical examination, ultrasound examination, and evaluation of semen (performed by G.C.W.E.) as part of routine (annually or more frequent) breeding soundness examinations for male dogs on the breeding programme. A positive diagnosis was made when the gland was increased in size and had intra-prostatic cysts, and there were erythrocytes in the third fraction of the ejaculate. Where the inbreeding coefficient was zero, implying no common ancestry of a dog’s parents, the value was set to missing, since this reflected limitations in the pedigree data rather than actual complete absence of any parental common ancestry.

TNLS and MOT were analysed using mixed linear models in ASREML-R version 4.2 [30]. The general form of the model was:

Y = Xb + Za + Wc + e

where Y is the vector of observations; W, X, and Z are known incidence matrices; b is the vector of fixed effects; a is the vector of random additive genetic effects with the distribution assumed to be multivariate normal (MVN) with parameters (0, Aσ^2^_a_); c is the vector of random permanent non-genetic effects of each individual distributed MVN with parameters (0, Iσ^2^_c_); and e is the vector of residuals distributed (0, Iσ^2^_e_). I represents an identity matrix of the appropriate size, A is the additive genetic relationship matrix, and σ^2^ denotes the variance of each of the random effects.

Estimates of variance components (σ^2^_a_, σ^2^_c_, σ^2^_e_) were used to calculate parameters. The phenotypic variance was the sum of all variance components (σ^2^_p_ = σ^2^_a_ + σ^2^_c_ + σ^2^_e_). The [narrow sense] heritability (h^2^) was calculated as the proportion of phenotypic variance that was additive genetic variance (σ^2^_a_/σ^2^_p_), and the permanent environment effect (c^2^) as the proportion of phenotypic variance that was permanent non-genetic variance (σ^2^_c_/σ^2^_p_). The repeatability (R) was calculated as R = h^2^ + c^2^ and is the proportion of variance that is due to permanent differences between individuals (both genetic and environmental [31]). Therefore, 1-R describes the environmental variation specifically due to temporal or localised circumstances across repeated measures across individuals. Statistical significance of h^2^ and c^2^ was confirmed using likelihood ratio tests.

For MOT and TNLS, age in months at collection (agem) and inbreeding coefficient (F) were included as covariates, and year of evaluation (yoe) and presence/absence of a diagnosis of BPH were included as fixed effects. Temporal trends, corrected for genetic and permanent environment effects, were calculated by regression of the individual effects of yoe determined by the mixed linear model on respective year.

### 2.2. Cryptorchidism

Records of incidences of unilateral and bilateral cryptorchidism (persisting to beyond 6 months of age) among all male puppies born in the assistance dog breeding programme between 1 January 1994 and 31 December 2023 were collated. Externally bred dogs were excluded. Individuals that were Labrador or Golden Retriever, or any mix of only these two breeds, were retained. From these data, rates of unilateral, bilateral, and total [unilateral + bilateral] cryptorchidism per year of birth were calculated. Trends were determined via simple linear regression over year of birth.

Rates of cryptorchidism (p) over the entire 30-year period were determined as number of uni- or bilaterally cryptorchid puppies (n_crypt_)/total number born (n). The variance (s^2^ = p [1 − p]), standard deviation (s), standard error of the mean (s.e. = s/√n), and 95% confidence intervals (95CI = 1.96 × s.e.) for observed values were calculated.

Whole period rates of cryptorchidism were compared across Labrador Retrievers (L), Golden Retrievers (GR), and first-generation crosses between L and GR (F1). Departure of the rate in F1 from the mid-breed mean would be indicative of heterosis (hybrid vigour). A chi-square test was used to determine the significance of differing rates of cryptorchidism among breed cohorts. Of the rates seen among breed cohorts, 95% confidence intervals were calculated as above.

Cryptorchidism was analysed using mixed linear models in ASREML-R version 4.2 [30] for individual breed cohorts: L, GR, and all crosses between the two excluding ‘pure’ animals (GRLX). The y-variable was a transformed value of the number of undescended testes (0/1/2) based on the whole period incidence using a truncated normal distribution, on the assumption that this more closely reflected presumed normal distribution of underlying genetic effects. The general form of the model was as described above, but substituting ‘Vi’ representing litter for ‘Wc’ representing permanent environment since there was only one observation per individual, but potentially multiple males per litter:

Y = Xb + Za + Vi + e

V is a known incidence matrix, and i is the vector of effects of each litter distributed MVN with parameters (0, Iσ^2^_i_). Thus, phenotypic variance was calculated as σ^2^_p_ = σ^2^_a_ + σ^2^_i_ + σ^2^_e_, and the litter effect as σ^2^_i_/σ^2^_p_. Inbreeding coefficient (F) was included as a covariate and year of birth (yob) as a fixed effect. For GRLX, the proportion of L breed was also included as a covariate. Analyses were repeated using the observed number of undescended testes (0/1/2) as the y-variable to determine temporal trends, corrected for genetic and litter effects, with the observed scale aiding interpretation. Temporal trends were calculated by regression of the fixed effects of yob determined in the mixed linear model on respective year and compared to those from the simple linear regression above.

Data preparation and analysis was performed in MS excel and R version 4.3.1 [32], using the specific package truncnorm version 1.0-9 [33] to determine truncated normal distribution values.

## 3. Results

### 3.1. Semen Quality Traits

There were 1468 semen evaluation records from 186 unique L males, and 1051 from 113 unique GR males. Within each year of evaluation, there were samples from a minimum of 30 unique L and 20 unique GR dogs. Samples came from dogs born between October 1996 and October 2022 (L), and September 1997 and November 2022 (GR), from dogs between 11.6 and 117.3 (L) and 12.0 and 119.8 (GR) months of age.

Samples from GR males tended to be collected from slightly older dogs (mean agem 50.87 vs. 47.67), with higher F (mean 0.076 vs. 0.062), and more often from those affected with BPH (12.94% of samples vs. 7.97%) than samples from L males.

Samples from L dogs tended to have higher MOT (mean = 70.54%, s.d. = 14.52, IQR = 65–80%) than GR (mean 65.58%, s.d. 17.31%, IQR = 60–75%). Samples from L males tended to have higher TNLS (mean = 72.56%, s.d. = 18.64%, IQR = 68–85%) than GR males (mean = 67.56%, s.d. = 21.33, IQR = 60–83%).

#### Genetic Analysis

The determined parameters and effects on MOT and TNLS in L and GR are shown in Table 1.

The heritability of MOT was estimated at 0.476 (s.e. 0.133) in GR, but in L was bound to zero. Conversely, the permanent environment effect of MOT was estimated at 0.296 (s.e. 0.073) in L, but was bound to zero in GR. Resultant repeatability of MOT in L was 0.296 (s.e. 0.036) and in GR was 0.476 (s.e. 0.056).

The heritability of TNLS was estimated at 0.372 (s.e. 0.128) in GR, and at 0.076 (s.e. 0.092) and not significantly different from zero in L. Conversely, the permanent environment effect of TNLS was estimated at 0.382 (s.e. 0.088) in L, and at 0.051 (s.e. 0.096) and not significantly different form zero in GR. Resultant repeatability of TNLS was more comparable across the two breeds than for MOT, being 0.458 (s.e. 0.037) in L and 0.423 (s.e. 0.044) in GR.

Thus, both traits appeared to have heritability estimates greater than zero in GR but permanent environment effects not significantly different to zero, and vice versa in L. For TNLS, the magnitude of estimates of h^2^ for L and c^2^ for GR were larger than zero (even though not statistically determinably so), whereas for MOT, the variance estimates (and so effects) were bound to zero in the model.

The covariate age (in months, agem) had a similarly negative effect on TNLS in both L and GR (−0.131, s.e. 0.020; −0.137, s.e. 0.029), equivalent to a decline of −1.58% and −1.64% over a year. For MOT, there was a negative effect of age (in months) determined in the GR (−0.080, s.e. 0.024), equivalent to a yearly decline of −0.97%. The estimated effect was not significantly different to zero in L (−0.029, s.e. 0.016). Inbreeding coefficient (F) had a significant negative effect on MOT and TNLS in the GR (−100.9, s.e. 39.8; −98.9, s.e. 46.2), equivalent to a decline of −12.6% MOT and −12.4% TNLS with every 0.125 increment in F (the value of F in the offspring of a half sib mating, with no further parental common ancestry). The estimated effect of F in L were smaller in magnitude (MOT −10.3, s.e. 20.3; TNLS −57.9, s.e. 32.5) and not significantly different to zero.

The effect of a diagnosis of BPH had a universally detrimental effect on traits: for MOT −5.96% (s.e. 1.66) in L and −5.35% (s.e. 1.722) in GR, and for TNLS −7.12% (s.e. 1.87) in L and −9.33% (2.11) in GR. Year of evaluation had a significant effect on TNLS in both breeds, and MOT in GR only. The results of regression of determined effect on year are shown in Table 2.

There was no determinable temporal trend in MOT in the L. In the GR, there was a significant reduction in MOT over time, equivalent to −4.76% over a decade. There was a similar decline detected in TNLS for GR, equivalent to −5.12% over a decade. However, the detected trend in TNLS in L was actually positive, equivalent to +3.5% over a decade.

### 3.2. Cryptorchidism

**Figure 1:**
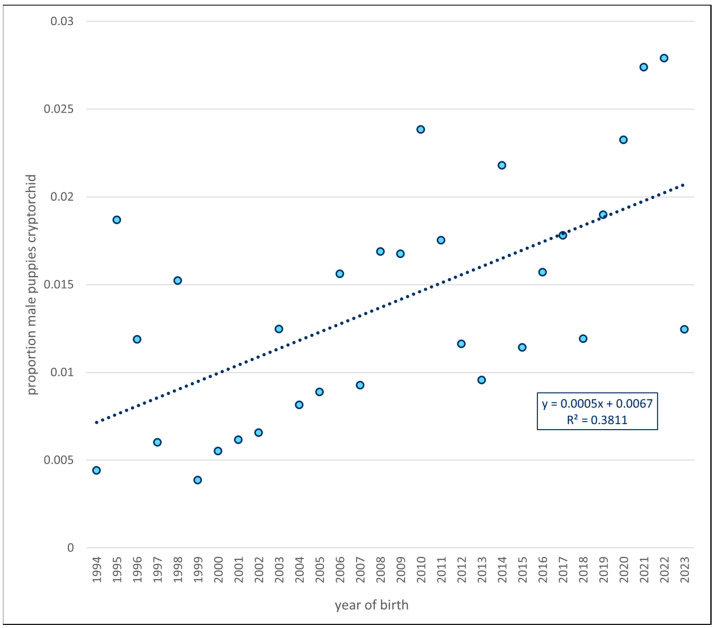
Plot of the incidence of unilateral and bilateral cryptorchid puppies born per year 1994–2023. The trend line (dashed) indicates a significant, increasing trend (*p* < 0.001), as denoted in the displayed equation. The R^2^ indicates that 38% of the variation in yearly incidence of cryptorchidism is accounted for by the temporal trend.

There were 5431 L, 1711 GR, and 8153 GRLX male puppies born between 1994 and 2023 inclusive (total 15,295). The total number of unilaterally cryptorchid males was 186, and bilaterally cryptorchid 24, summing to 210 in total, an overall incidence of 1.37% over the 1994–2023 period. The total proportion of male puppies that were unilaterally or bilaterally cryptorchid over year of birth is shown in Figure 1. The regression coefficient of the proportion of cryptorchid males on year of birth was 4.7 × 10^−4^ (*p* = 0.00028), or 0.047% per year, and equivalent to an increase of 1.4% over the 30-year period.

The number of males born, the number unilaterally and bilaterally cryptorchid, the total, and respective percentages of total born per year within each breed cohort (L, GR, GRLX) is given in the Supplementary Materials. The results of regression of incidence of cryptorchidism on year of birth individually for each breed cohort are shown in Table 3.

Statistically significant increasing trends were discerned for L (0.00083, *p* < 0.01) and GRLX (0.00046, *p* < 0.001). However, the regression coefficient was small (4.12 × 10^−5^) and not statistically different to zero (*p* > 0.1) in the GR.

#### 3.2.1. Genetic Analysis

Linear models of transformed number of undescended testes revealed no determinable heritability in any of the breed cohorts (estimates of σ^2^_a_ were bound to zero). There was evidence of small and significant litter effects in L (0.042, s.e. 0.012) and in GR (0.063, s.e. 0.022), but not in the GRLX (0.024, s.e. 0.018). Inbreeding coefficient was determined to have a significant effect in L only (0.178, s.e. 0.070 on the observed scale, equivalent to an increase in risk of 0.022 or 2.2% per 0.125 increase in F). The effect of proportion of L in GRLX was not significant (−0.045, s.e. 0.051). The temporal trends from regression of corrected effects of year of birth individually for each breed cohort are shown in Table 4.

Statistically significant increasing trends were discerned for L (0.00106, *p* < 0.001) and GRLX (0.00077, *p* < 0.01), but not GR (8.50 × 10^−5^, *p* > 0.1). Discerned trends in L and GRLX were larger in magnitude than observed from regression of raw, uncorrected data (Table 3).

#### 3.2.2. Evidence for Heterosis

**Figure 2:**
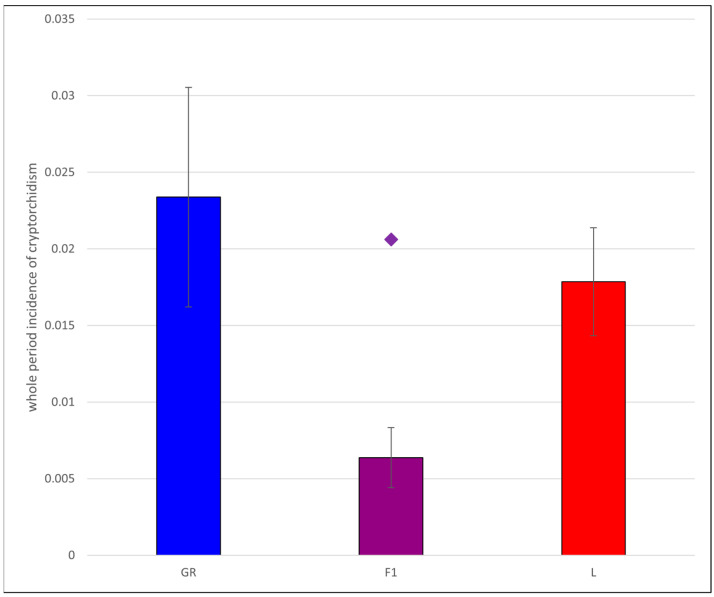
Bar plot of the whole period incidence of cryptorchidism for Golden Retriever (GR, blue), Labrador Retriever (L, red), and first generation cross (F1, purple) male puppies. Error bars are 95% confidence intervals (+/−1.96 × standard error of the mean) and show that incidence among F1 is significantly lower than either GR or L, and deviates from the mid-breed average of 0.02062 (shown as a diamond).

For L, the whole period incidence of cryptorchidism was 1.79% (s.e. = 0.180%), and for GR, 2.34% (s.e. = 0.365%), making the mid-breed mean incidence 2.06%. There were 40 of 6263 F1 puppies with cryptorchidism, an incidence of 0.64% (s.e. = 0.100%). A chi-square test determined that observed numbers deviated significantly from expected (*p* = 1.57 × 10^−10^), implying that there are breed differences in incidence of cryptorchidism over the 30-year period. These results are displayed graphically in Figure 2, with the significantly lower incidence among F1 puppies than either L or GR providing evidence of heterosis in this trait.

## 4. Discussion

This study has confirmed continuing detrimental temporal trends in important semen quality traits in Golden Retrievers and in rates of cryptorchidism in Labrador Retrievers in an assistance dog population. The effects amount to a decline in progressive, fast, forward motility of −4.76% and in total number live sperm of −5.12% in Golden Retrievers, and an increase in rate of cryptorchidism in Labrador Retrievers of +1.06% over a decade. Were these trends to continue in a linear fashion, it would cause considerable concern for this assistance dog breeding programme. In addition, and to the authors’ knowledge for the first time, the study has reported pedigree-derived heritability estimates of two semen quality traits, fast, forward progressive motility and total normal live sperm, in a large population of Golden Retrievers.

The semen quality trends determined in this study are consistent with those reported previously. Lea et al. [11] observed a decline in MOT of 30% over 26 years from 1988 to 2014 in the same population. The magnitude of the trend detected in GRs between 2006 and 2023 inclusive here is somewhat less, equivalent to −12.5% (and −13.3% for TNLS) over a 26-year period but is corrected for other effects. Similarly, Lea et al. [11] reported an increasing trend in the incidence of cryptorchidism, and here we report an overall increase of +0.47% per decade determined between 1994 and 2023 inclusive for all L, GR and crosses, and breed-specific corrected 10-year trends of +1.06% in L and +0.77% in GRX. The results from this study therefore add to the evidence of extant temporal changes in canine testicular and fertility traits in this population.

The changes observed in this assistance dog population appear to be in line with wider trends in semen quality traits across species and throughout the world. Harris et al. [13] report declines in progressive motility in horses of −0.96% annually between 1984 and 2019, and Wahl and Reif [12] reported declines in concentration and TNLS in US Holstein bulls from 1965 to 1995. However, it is humans that are the most popular subject of research in this area, with multiple studies determining and comparing trends across geographical regions. In a recent meta-analysis, Levine et al. [14] determined worldwide declines in human sperm count and concentration, noting that the trend appeared to be accelerating. Semen traits, however, are just one aspect of a wider raft of male reproductive disorders, including testicular cancers, hypospadias, and cryptorchidism, which appear to be increasing and have been termed Testicular Dysgenesis Syndrome [8]. The rate of observed changes and geographical variation has led to the hypothesis that environmental chemicals (ECs) are major drivers of these declining trends. Sumner et al. [10] determined regional variation in profiles of testicular ECs and parallel observations of perturbed testicular morphology in dogs. The same ECs (polychlorinated bisphenyls [PCB] and diethylhexyl phthalate [DEHP]) had previously been shown to have a detrimental effect on sperm function [9]. For cryptorchidism, too, there is growing evidence that an increase in incidence is influenced by prenatal exposure to ECs which disrupt hormone production and function, upon which testicular descent is dependent [34]. It is thought that these effects are due to a combination of exposures rather than a single chemical [8]. Sumner et al. [9] showed that exposure to two chemicals had a similar effect on semen traits (reducing motility) in both the human and dog, implying a common aetiology and suggesting the dog as a sentinel species. The same authors, in a review discussing the dog as a sentinel species for environmental effects on human fertility [35], note that “both pre- and post-natal stages of early life development are sensitive to chemical exposures and that such changes could potentially cause long term effects in the adult,” with the weight of evidence suggesting a common aetiology involving a complex group of ECs. Therefore, it appears reasonable to suggest that the trends in semen traits and cryptorchidism observed in this population of Labrador and Golden Retrievers share a common cause, thought to be related to exposure to ECs, with those observed in other species, particularly humans with whom dogs have a common environment.

An interesting result from the present study was the difference in detected trends across different breeds in this population: the decline in semen quality apparent in Golden but not in Labrador Retrievers, and the rise in cryptorchidism seen in Labrador but not in Golden Retrievers. There was no management or environmental variation confounded with breed in this study; in fact, it may be suggested that environment and management was more uniform than would be expected in canine field studies due to the consistent procedures and protocols and provision of equipment by the assistance dog organisation. There appeared to be general breed differences apparent in both the semen quality traits and cryptorchidism rates, with Labrador Retrievers generally being superior to Golden Retrievers (mean MOT = 70.5% vs. 65.6%; mean TNLS 72.6% vs. 67.6%; cryptorchidism 1.8% vs. 2.3%). The Golden Retrievers tended to be slightly older (mean agem 50.87 vs. 47.67), more inbred (mean F 0.076 vs. 0.062), and had a higher incidence of BPH (12.94% of samples vs. 7.97%) than Labrador Retrievers, which may have had an influence on the overall comparison of semen quality traits. However, such breed differences could also easily be influenced by inherent genetic differences between the two breeds. The development of modern dog breeds has bequeathed particular genetic features, including long stretches of uniformity in sequence within, but that may differ between, breeds [36] and that may account for inter-breed phenotypic variation. Assuming there are such genetic differences between breeds in this population, the differences in the determined temporal trends in semen quality traits and in rates of cryptorchidism between Labrador and Golden Retrievers could therefore point to genetic by environmental interactions. A genetic by environment interaction is a differential effect on phenotypes of genetics across environments (for example, a modified effect of a genetic variant on obesity when diet is unrestricted [37]). Here, the presumed environmental influences driving observed temporal changes (posited to be exposure to ECs), which are randomly applied across the two breeds, have differential phenotypic outcomes depending on underlying genetics, which are stratified across breed. The physiological processes underlying these traits, influenced by proteins coded for at such genomic locations, may therefore be susceptible to particular environmental factors in one breed but not the other. If researchers were able to locate such genes, with variants conferring susceptibility or immunity to testicular dysgenesis from environmental influences, they could be useful candidates for studies in humans. The breed differences in the rate and trend in cryptorchidism mean that while Golden Retrievers currently display a higher incidence of cryptorchidism, Labrador Retrievers are catching up. In both semen quality traits, the Golden Retrievers tended to have lower levels than Labrador Retrievers and were declining, whereas the Labrador Retrievers showed no discernible trend in motility but actually seemed to be experiencing an increase in total number live sperm, so the gap is widening.

The differences in semen quality traits between breeds extended to the partitioning of variance, with no detectable heritability in either motility or TNLS in the Labrador Retriever, in contrast to sizeable and significant estimates in the Golden Retriever. This pattern was reversed for the permanent environment effect. The permanent environment is circumstance that has a consistent effect on a phenotype across measures at repeated timepoints for an individual. It differs between individuals and is unique to each individual, unlike genetics, which is [partially] shared by relatives. A pertinent example might include a prepubertal illness that has a lifelong impact on sperm motility, or indeed, pre- or postnatal exposure to ECs (see above) that similarly impacts sperm quality over a lifetime. The ability to detect heritability and permanent environment will depend on a number of factors, including the extent of genetic variation within a population and the number of repeated measures per individual (with a single measure, it is impossible to separate permanent environment from environmental effects specific to the particular timepoint). The presence of repeated measures enables the calculation of repeatability, and 1-repeatability describes the variance due to environment specific to temporal or spatial circumstances [31]. The repeatability of TNLS was similar across breeds at 0.42 (GR) and 0.46 (L), despite differences in its composition. For MOT, the repeatability was higher in GR (0.48) than L (0.30) and respective non-significant components were both bound to zero, suggesting difficulty in the model partitioning additive genetic from permanent environment variances. The detectable repeatabilities for semen traits imply that, while there is much variation due to specific environmental effects, there is some degree of consistency among measures of semen traits in these populations, and that repeated measures improve the accuracy of predicted values.

The heritability estimates for cryptorchidism reported here were not determinable from zero. Previously, an estimate of 0.23 had been reported in Dutch Boxers by Nielen et al. [38]. Estimates of heritability are population-specific, however, and the prevalence of cryptorchidism reported by Nielen et al. [38] was much higher than observed here (10.7% vs. 1.8% for Labrador Retrievers and 2.3% for Golden Retrievers) and so had a much greater phenotypic variance. The results here imply that within these breed populations there was no detectable additive genetic variance in cryptorchidism. However, examination of the incidence of cryptorchidism between these two breeds and crosses revealed a significant deviation in the F1 cross from the mid-breed mean of Labrador and Golden Retriever, providing evidence of heterosis, or hybrid vigour. Heterosis is a result of differential genetic variants ‘fixed’ in either breed due to selection or drift. The resulting ‘hybrid vigour’ of the F1 is thought to be due either to dominance of alleles from one breed over mildly deleterious alleles from the other cumulatively over many genes, or overdominance where the heterozygote performance exceeds that of either homozygote [39]. Either way, implicit to the observation of heterosis is a lack of genetic (allelic) variation within each parental breed and allelic dominance rather than additivity, both consistent with the lack of detectable additive genetic variation and so heritability. The detection of heterosis implies inbreeding depression in the parent breeds, which again is a function of allelic dominance rather than additivity [31]. The superior performance of cross vs. pure breeds has been reported in dogs for a number of traits previously [40]; however, the authors note that difficulties in classification of mixed or cross breed dogs as true first generation (F1) crosses hinders the definitive identification of heterosis, rather than potentially simply reflecting differential prevalence across breed groups [41,42,43,44,45,46]. As such, the detailed pedigree records maintained by a breeding programme incorporating crosses, as in the present study, is hugely useful in confirming the presence of heterosis and so, by extension, inbreeding depression. Such data have been utilised before to identify crossbreeding parameters in puppy behavioural traits [47], although estimates of heterosis in this case were not statistically significant. Further research utilising pedigree data with well-defined crosses to ascertain breed effects, heterosis, and recombination loss in a range of health and behavioural traits would be of great interest.

Beyond the genetic and temporal trends discussed, the present study determined modest but significant negative effects of BPH on semen quality traits in both breeds. There is little evidence of such effects having been examined before, but Gonzalez et al. [48] found no impact of drugs used to treat BPH (anastrozole and tamoxifen) on sperm volume, count, motility, and morphological abnormalities in dogs or in humans. Hoover and Naz [49] reported no induction of immunity via antibodies to spermatozoa or seminal components in men with BPH. Therefore, the nature of the reduction in semen quality when BPH is present is unknown, although it is plausible that there are changes to the nature of the prostatic fluid which at ejaculation affects the sperm.

The present study also reported detrimental effects of inbreeding on semen traits in GR and cryptorchidism in L. There are multiple studies that have reported inbreeding depression in semen traits in pigs [50], bulls [51], sheep [52], and dogs [53], but none to our knowledge on cryptorchidism. The decline in semen quality traits with age determined here are in line with established trends in both the dog and other species [54].

## 5. Conclusions

In conclusion, this study has reaffirmed detrimental temporal trends in traits indicative of testicular dysgenesis in this population, in line with declines noted in human male fertility and with a presumed aetiology involving pre- and post-natal exposure to ECs. Interestingly, we noted different temporal trends in different breeds, implying genetic by environment interactions whereby exposure to ECs has a different physiological manifestation dependent on differential genetics stratified with breed. While semen quality traits were declining in the Golden but not the Labrador Retriever, rates of cryptorchidism were rising in the Labrador but not the Golden Retriever. In addition, we presented evidence of heterosis in cryptorchidism, with a far lower incidence among F1 males than either Labrador or Golden Retriever males. These results demonstrate the utility of detailed phenotypic and pedigree records from managed canine populations in determining and characterising such trends and features, and which may assist in research into the underlying genetic causes of similar traits in humans.

## Figures and Tables

**Table 1:** Effects on %motility (MOT) and %total normal live sperm (TNLS) determined in Labrador Retrievers (L) and Golden Retrievers (GR) from mixed model analysis, including standard error (s.e.) and notation of statistical significance (sig; n.s. = not significant, * = *p* < 0.05, ** *p* < 0.01, *** = *p* < 0.001). Heritability (h^2^) and permanent environment effect (c^2^) sum to the repeatability (R). Fixed effects are year of evaluation (yoe) and diagnosis with benign prostatic hyperplasia (bph), and covariates are age in months at collection (agem) and inbreeding coefficient (F).

		L			GR		
		Effect	s.e.	Sig	Effect	s.e.	Sig
MOT	h^2^	1 × 10^−7^	0.068	n.s.	0.476	0.133	**
	c^2^	0.296	0.073	**	2.43 × 10^−7^	0.095	n.s.
	R	0.296	0.036	***	0.476	0.056	***
	yoe	0.014	0.141	n.s.	−0.476	0.181	*
	agem	−0.029	0.016	n.s.	−0.080	0.024	**
	F	−10.3	20.3	n.s.	−100.9	39.8	*
	bph	−5.961	1.656	***	−5.346	1.722	**
TNLS	h^2^	0.076	0.092	n.s.	0.372	0.128	**
	c^2^	0.382	0.088	***	0.051	0.096	n.s.
	R	0.458	0.037	***	0.423	0.044	***
	yoe	0.350	0.132	*	−0.512	0.171	**
	agem	−0.131	0.020	***	−0.137	0.029	***
	F	−57.9	32.5	n.s.	−98.9	46.2	*
	bph	−7.115	1.866	**	−9.325	2.107	***

**Table 2:** Results of simple linear regression of determined effects of year of evaluation on %motility (MOT) and %total normal live sperm (TNLS) from linear models corrected for genetics and permanent environment effects on year for Labrador Retrievers (L) and Golden Retrievers (GR). Shown are the regression coefficient (b), standard error (s.e.), *p*-value and notation of statistical significance (n.s. = not significant, * = *p* < 0.05, ** *p* < 0.01), the low and high 95% confidence intervals (95CI), and the projected percent change over a decade.

		b	s.e.	*p*-Value		lo 95CI	hi 95CI	% over 10 Years
MOT	L	0.01387	0.14053	0.92263	n.s.	−0.2841	0.31178	0.14
	GR	−0.47599	0.18112	0.01827	*	−0.86	−0.092	−4.76
TNLS	L	0.35037	0.13185	0.01721	*	0.07085	0.62988	3.50
	GR	−0.51233	0.17092	0.00853	**	−0.8747	−0.15	−5.12

**Table 3:** Results of simple linear regression of incidence of cryptorchidism on year of birth for Labrador Retrievers only (L), Golden Retrievers only (GR), crosses between L and GR (GRLX), and all breed cohorts (All). Shown are the regression coefficient (b), standard error (s.e.), *p*-value and notation of statistical significance (n.s. = not significant, ** *p* < 0.01, *** = *p* < 0.001), the low and high 95% confidence intervals (95CI), and the projected percent change in incidence over a decade.

	b	s.e.	*p*-Value		lo 95CI	hi 95CI	% Change 10 Years
All	0.00047	0.000113	0.0003	***	0.00024	0.00070	0.47%
L	0.00083	0.000229	0.0011	**	0.00036	0.00130	0.83%
GR	4.12 × 10^−5^	0.000428	0.9240	n.s.	−0.00084	0.00092	0.04%
GRLX	0.00046	0.000115	0.0004	***	0.00022	0.00070	0.46%

**Table 4:** Results of simple linear regression of determined effects of year of birth from linear models corrected for genetics and litter on year of birth for Labrador Retrievers only (L), Golden Retrievers only (GR), crosses between L and GR (GRLX). Shown are the regression coefficient (b), standard error (s.e.), *p*-value and notation of statistical significance (n.s. = not significant, ** *p* < 0.01, *** = *p* < 0.001), the low and high 95% confidence intervals (95CI), and the projected percent change in incidence over a decade.

	b	s.e.	*p*-Value		lo 95CI	hi 95CI	% Change 10 Years
L	0.00106	0.000268	0.0005	***	0.00051	0.00161	1.06%
GR	8.50 × 10^−5^	0.000547	0.8775	n.s.	−0.00103	0.00121	0.09%
GRLX	0.00077	0.000211	0.0010	**	0.00034	0.00121	0.77%

## Data Availability

Restrictions apply to the availability of these data. Data were obtained from Guide Dogs UK and are available from Rachel Moxon with the permission of Guide Dogs.

## Supplementary Materials

The following supporting information can be downloaded at: https://www.mdpi.com/article/10.3390/ani15142073/s1, Table S1: The number of males born, the number unilaterally and bilaterally cryptorchid, the total, and respective percentages of total born per year within each breed cohort.

## Abbreviations

The following abbreviations are used in this manuscript:

agem	Age in months
BPH	Benign prostatic hyperplasia
F	Inbreeding coefficient
F1	First generation cross (between two breeds)
GR	Golden Retriever
GRLX	Golden Retriever and Labrador mix, excluding ‘pure’ breeds
IQR	Inter quartile range
L	Labrador Retriever
MVN	Multivariate normal distribution
MOT	% sperm with fast forward progressive motility
s.e.	Standard error of the mean
TDS	Testicular dysgenesis syndrome
TNLS	Total number of live sperm
yoe	Year of evaluation
